# The impact of a pharmacist on post-take ward round prescribing and medication appropriateness

**DOI:** 10.1007/s11096-018-0775-9

**Published:** 2019-01-04

**Authors:** B. Bullock, P. Donovan, C. Mitchell, J. A. Whitty, I. Coombes

**Affiliations:** 1 Pharmacy Department, Royal Brisbane and Women’s Hospital, Cnr Butterfield St and Bowen Bridge Rd, Herston, QLD 4029 Australia; 2 School of Pharmacy, University of Queensland, Pharmacy Australia Centre of Excellence, Level 4, 20 Cornwall Street, Woolloongabba, QLD 4102 Australia; 3 Medical Education Unit, Gold Coast Hospital and Health Service, 1 Hospital Blvd, Southport, QLD 4215 Australia; 4 School of Medicine, University of Queensland, Level 5, Building 69, St Lucia, QLD 4072 Australia; 5 Department Clinical Pharmacology, Royal Brisbane and Women’s Hospital, Cnr Butterfield St and Bowen Bridge Rd, Herston, QLD 4029 Australia; 6 Norwich Medical School, University of East Anglia, Norwich Research Park, Norwich, NR4 7TJ UK

**Keywords:** Australia, Communication, Medication safety, Prescribing, Team work, Ward round

## Abstract

*Background* Medication communication and prescribing on the post-take ward round following patient admission to hospital can be suboptimal leading to worse patient outcomes. *Objective* To evaluate the impact of clinical pharmacist participation on the post-take ward round on the appropriateness of medication prescribing, medication communication, and overall patient health care outcomes. *Setting* Tertiary referral teaching hospital, Brisbane, Australia. *Method* A pre-post intervention study was undertaken that compared the addition of a senior clinical pharmacist attending the post-take ward was compared to usual wardbase pharmacist service, with no pharmacist present of the post-take ward round. We assessed the proportion of patients with an improvement in medication appropriateness from admission to discharge, using the START/STOPP checklists. Medication communication was assessed by the mean number of brief and in-depth discussions, with health care outcomes measured by comparing length of stay and 28-day readmission rates. *Main outcome measures:* Medication appropriateness according to the START/STOPP list, number and type of discussions with team members and length of stay and readmission rate. *Results* Two hundred and sixty patients were recruited (130 pre- and 130-post-intervention), across 23 and 20 post-take ward rounds, respectively. Post-intervention, there was increase in the proportion of patients who had an improvement medication appropriateness (pre-intervention 25.4%, post-intervention 36.9%; *p* = 0.004), the number of in-depth discussions about patients’ medication (1.9 ± 1.7 per patient pre-intervention, 2.7 ± 1.7 per patient post-, *p* < 0.001), and the number relating to high-risk medications (0.71 ± 1.1 per patient pre-intervention, to 1.2 ± 1.2 per patient post-, *p* < 0.05). Length of stay and 28-day mortality were unchanged. *Conclusion* Clinical pharmacist participation on the post-take ward round leads to improved medication-related communication and improved medication appropriateness but did not significantly improve health care outcomes.

## Impacts on practice

- Pharmacist input to medical officers decision making during a patients’ admission can improve the appropriateness of medications for patients.
- Clinical pharmacists input improves the collaborative optimisation of medication use. The involvement of a pharmacist can ensure increased communication about medications during the admission process and that decisions made by medical staff around medication prescribing are enacted upon.
- This model of care ensures the efficient use of all members of the healthcare team by ensuring pharmacists input to optimising medicines in conjunction with senior medical staff. Although this study was not powered to show impacts on patient health care outcomes (such as length of stay and unplanned readmissions), the addition of a pharmacist on the post take ward round ensures more appropriate medication use, which is essential for an ageing population with complex conditions and a high medication burden.

## Introduction

Poor prescribing leads to patient harm through increased medication errors and adverse drug events (ADEs) [1]. Interventions by clinical pharmacists are known to improve the safety and quality of prescribing [2–5], with most interventions occurring independent of the ward round [6, 7]. In Australia, the duties of hospital based clinical pharmacists working on hospital wards includes medication reconciliation, clinical review and overall contribution to the medicines management pathway in order to minimise the risks associated with the use of medicines and to optimise the use of medicines [6].

In many Australian public hospitals, patients are initially seen by junior medical staff on admission who undertake assessment and institute preliminary management plans. The patient is then presented to the treating consultant on a ward round often termed the “post-take ward round” (PTWR), which usually occurs on the day of or day after presentation. On the PTWR the treating consultant will often make diagnoses, refine management plans and begin attempts to optimise the patient’s pharmacotherapy [8]. The PTWR is usually only attended by senior (consultant) and junior medical staff (resident and registrar, sometimes referred to as house officers). Our initial study of medication communication on the PTWR without a pharmacist present found that the level of medication communication and implementation of agreed medication-related management decisions was suboptimal [9].

Pharmacist participation on ward rounds influences the quality of prescribing and may lead to better outcomes than pharmacist interventions at other times [10, 11] with prior studies identifying improved rates of prescribing errors and preventable ADEs [7, 12, 13]. However, there is limited evidence of benefits of a pharmacist attending the PTWR. A study by Fertleman et al. [2] showed that a pharmacist on a general medical PTWR, in comparison to normal practice whereby pharmacist input occurred after the PTWR, resulted in improved accuracy of drug history documentation, reduced prescribing costs and a decrease in potential medication risk to the patient. However, this was a small study (103 patients) and used a single intervention pharmacist which potentially limits the generalisability of results. Additionally, the degree of communication between senior and junior medical staff and pharmacists was not specifically investigated nor was medication appropriateness assessed.

## Aim of the study

We aimed to evaluate the impact of clinical pharmacist participation on the PTWR on medication prescribing as measured by medication appropriateness, the level and extent of medication communication and patient and service delivery related outcomes, including length of stay.

## Ethics approval

Ethics approval was obtained from the Hospital and University Human Research Ethics Committees (HREC/13/QRBW/443;2014000705). Verbal consent was obtained and recorded for all patients and written consent was obtained from all pharmacists and medical staff prior to the period of observation. Consent could be withdrawn at any time.

## Method

### Study design

**Fig. 1:**
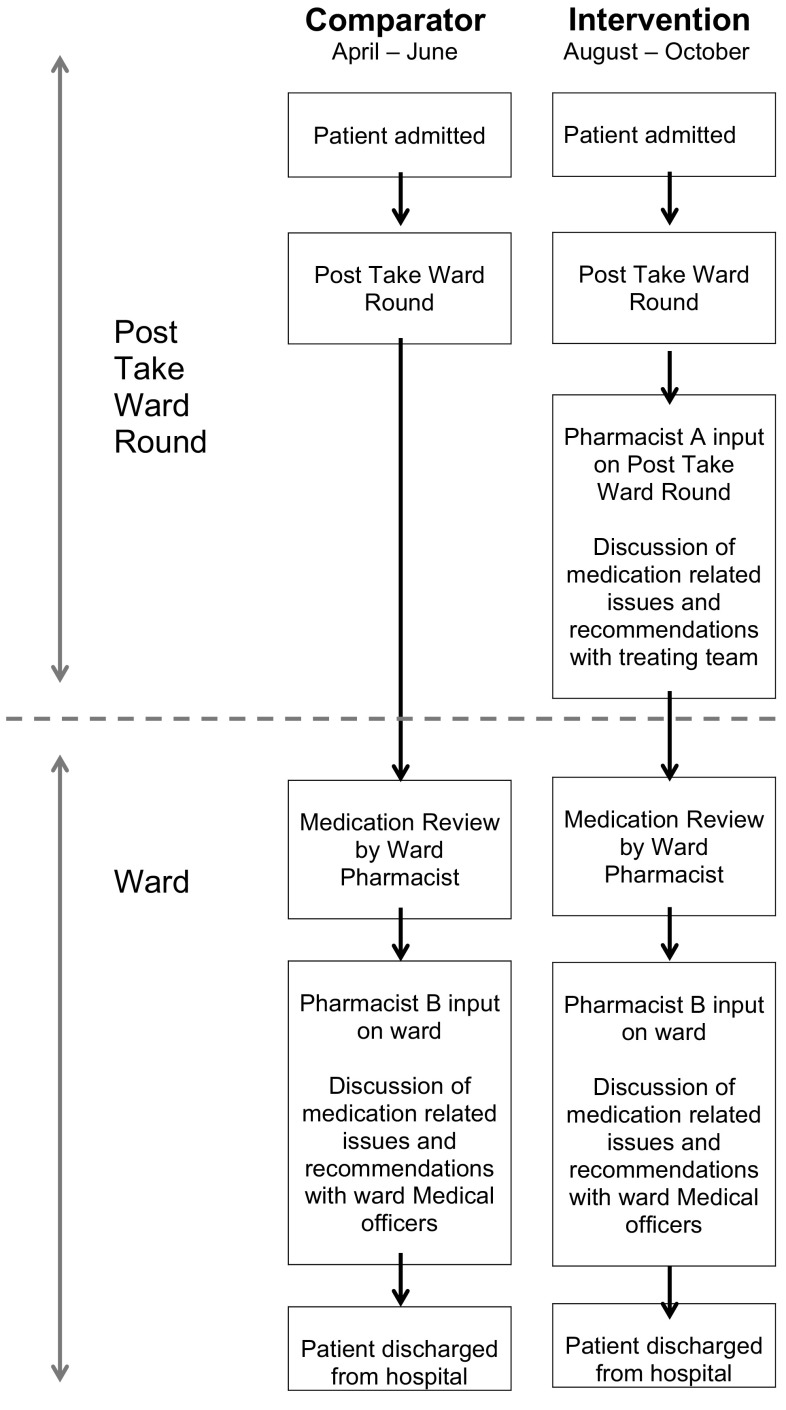
Flow chart of comparator and intervention phase of study

The study occurred in the Internal Medicine Department at a 929-bed quaternary and tertiary referral teaching hospital in Brisbane, Australia. The study was a pre-post intervention trial, with the initial observation period (comparator group) occurring over 6-weeks between April and June 2014. The comparator group consisted of usual medical care that included daily ward pharmacist review (including within 24 h of admission) on weekdays, with no pharmacist present on the PTWR. The intervention period occurred during a six-week period between August and October 2014 (intervention group). The intervention consisted of, in addition to usual medical care, a senior clinical pharmacist participating in the PTWR team (see Fig. 1). One of four pharmacists participated, each having a minimum of 6 years of clinical pharmacy experience, employed at a senior pharmacist level and had recently demonstrated a consistent level of competent ward-based performance measured using a clinical competency assessment tool [6]. The lead researcher briefed each of the pharmacists regarding their role and the expected level of interaction on the PTWR.

### Participants and data collection

Patients (18 years or older) under the care of all eight of the hospital’s internal medical teams were observed in both study phases. Inpatient medication order forms, the Discharge Medication Record (DMR) and medical records were used to collect data before the PTWR and on hospital discharge. The PTWR would start at 8 am and patients were included in the study if they were seen between 8 and 11 am for pragmatic reasons (availability of the research pharmacist). Patients seen during this time were thought to be a representative sample of the patients admitted overall as generally the team would review the patients starting with those most recently admitted as those were the patients admitted by the medical officer working overnight, allowing that medical officer to hand over patients and complete their shift in a timely manner. Although not random sampling, the same method of patient inclusion occurred in both the comparator and intervention groups.

One experienced pharmacist observed discussion related to individual patients for both groups. The observer did not participate in any discussions or prescribing decisions unless there was a need to intervene to prevent or highlight a potential adverse event that had so far not been realised and may cause immediate harm. Patient demographic information, reason for admission classified according to diagnosis related group (DRG), medical co-morbidities, details of staff present on PTWR, the total time for each patient consultation, medications on admission and discharge, evidence of inpatient clinical pharmacy review, discussion of previous allergy/ADR history and data on completion and provision of DMR were collected using a structured form. All medications were classified according to the anatomical therapeutic chemical (ATC) classification system.

Any mention of medication was recorded as medication related communication and was used to assess the frequency and depth of medication-related discussions. If there was a two-way discussion between medical staff and/or the pharmacist with the intention of the discussion as to whether a treatment modification was required, this was classified an in-depth discussion. If there was no two-way discussion, rather a brief mention of the medication by one person, this was classified as brief medication discussion (e.g., if a medication list was read out and there was no further discussion). Each medication was categorised as high risk (antibiotics, potassium, insulin and other hypoglycaemic agents, narcotics and other sedatives, chemotherapy and heparin and other anticoagulants) or non-high risk of medication related harm according to the Australian Safety and Quality Council’s APINCH classification system [14]. Further, for all medication-related discussions, it was assessed as to whether any proposed treatment modification was properly actioned (i.e., the change agreed upon during discussions was actioned).

In addition to investigating medication related discussion, medication appropriateness was assessed using the START/STOPP tools, with the START checklist a validated register of medications that may be appropriate to initiate, while the STOPP list identifies potentially inappropriate prescribing [15]. The lead researcher applied each of the START/STOPP criteria to the admission and discharge medications each patient was prescribed on the medication chart and using the patient medical history from the admission and discharge notes. If a patient was appropriately prescribed or de-prescribed a medication on these criteria lists, this would represent an improvement in medication appropriateness, with each indicator. A total score was given for each patient with the term score defined as the total number of flagged START/STOPP criteria. Each indicator was a yes/no and counted as one point. If there were cases of multiple indicators that applied to the same drug for a different reason, these were each counted as one. If there were multiple drugs which triggered the same indicator, this could only be scored once per indicator.

The START/STOPP tool was chosen due to its ease of use, inclusion of both potentially inappropriate medications and recommendations on medications which should be started, as it has been validated as an overall measure of medication appropriateness and is appropriate for the Australian setting [15, 16]. The START/STOPP tool was designed for use in patients 65 years or above and therefore the results in this patient subgroup were independently investigated; however it was also applied to all study patients as many of the criteria may be applicable to all adult patients, there is evidence of association with adverse outcomes across wider patient groups and its use in all adults would tend to under- rather than overestimate any benefits seen in this study [17].

The occurrence of a ward based clinical pharmacy review was measured by evidence of either (1) a pharmacist signature indicating a medication review had been completed and/or (2) the completion of a medication action plan (MAP), which is a document completed by a pharmacist during a medication review. Hospital related reporting data was collected retrospectively from hospital administration.

### Outcome measures

*Primary outcome* The difference in proportion of patients with an improvement in medication appropriateness from admission to discharge, between the comparator and intervention groups.

*Secondary Outcomes* The difference between the comparator and intervention groups for the following:

- The mean change in START/STOPP score* counted pre-PTWR and at discharge, with a pre-specified subgroup analysis of patients 65 years of age and older.
- Medication-related discussion on the PTWR (all discussions, plus in-depth discussions), with a subgroup analysis of high risk medications.
- For all in-depth medication related discussions, the difference in the number which were able to be actioned and that were actioned (i.e., led to a change in therapy) throughout the patients’ hospital stay.
- Patient and service delivery related outcomes:
  - Duration of the PTWR (e.g., time spent by PTWR team with each patient).
  - Ward based pharmacist activity.
  - Length of hospital stay.
  - Unplanned readmission to the same hospital at 30 days.

### Data analysis

It was difficult to undertake a valid sample size calculation given the scarcity of literature assessing the impact of a clinical pharmacist on PTWR on medication appropriateness and therefore the sample size was based on pragmatism using the maximum time available for the research staff to be able to undertake data collection. However, we predicted, given these time constraints, that we would be able to recruit ~ 125 patients in each group which is over double the size of the only other study to address a pharmacist on the PTWR [2, 3]. It was anticipated that, should significant results not be achieved with the sample, that these results could be used to estimate a sample size for a more definitive trial.

R commander version 3.2.4 (2016–03–10) was used for data analysis. Patient demographics and other continuous data are presented as mean ± standard deviation (or median and Inter quartile range where appropriate). Chi squared tests were used to compare differences in proportions. *T* tests were used to compare differences in means where data were normally distributed and nonparametric alternative (Wilcoxon rank sum) were used where data were not normally distributed. Categorical and binary data have been expressed as counts and percentages of the total number of possible outcomes. Statistical significance was achieved if *p* < 0.05.

## Results

During the 6-week observation period, 23 PTWRs with 130 patient consultations were observed in the comparator group and 20 PTWRs with 130 patient consultations in the intervention group. The characteristics of the comparator and intervention groups are shown in Table 1.

**Table 1:** Patient characteristics

	Comparator group (N = 130)	Intervention group (N = 130)	*p* Value
Gender (female) *N* (%)	61 (47%)	69 (53%)	0.32^*^
Age mean (± SD)	66 ± 19	63 ± 20	0.20^†^
Age > 65 *N* (%)	75 (58%)	67 (51.5%)	0.32^*^
Mean number of medications on admission, prior to PTWR mean, (SD)	8.9 ± 5	8.56 ± 4.47	0.54^†^
Mean number of medications on discharge mean, (SD)	8.2 ± 4.79	7.8 ± 5.07	0.03^†^

Statistical tests: * Chi squared, † student’s *T* test

There was no significant difference in the change in number of medications in the two groups from admission to discharge (mean reduction in baseline cohort − 0.69 ± 2.93, compared to comparator cohort 0.76 ± 3.71, *p* = 0.45).

Admission medications were similar across both groups according to their ATC classification, with the most common systems “Alimentary tract and metabolism” accounting for 23% of comparator and 24.7% of intervention admission medications, “cardiovascular system” accounting for 21.2% of comparator and 17.2% of intervention admission medications and “nervous system” accounting for 22.9% of comparator and 27% of intervention admission medications. The reasons for admission were also similar, with 60 individual DRG presentations in the comparator cohort: the most frequent classifications being “respiratory infection or inflammation” (10) and “chest pain” (7). In the intervention group, there were 70 individual DRG presentations with “syncope and collapse” (7) and “chest pain” (6) the most common. The DRG presentations seen are representative of a general medicine population.

There were no difference in the proportion of patients prescribed high-risk medications and the types and proportion of each type of high-risk medications was similar between groups (e.g., “heparin and other anticoagulants” which made up 35.5% in the comparator group and 40.8% in the intervention group and “antibiotics” made up 35.5% of in-depth discussions in the comparator group and 33.7% in the intervention group).

### Patient and medication communication

Table 2 shows the frequency of “in depth medication discussions” and shows a statistically significant greater number of medication discussions that were “in-depth” discussions with a mean of and 2.7 per patient in the intervention group and 1.9 per patient in the comparator group (*p* < 0.05). The number of discussions relating to high-risk medications significantly improved from a mean of 0.71 per comparator patient to 1.2 per intervention patient (*p* < 0.05).

**Table 2:** Patient and medication details discussed

	Comparator group N = 130 patients	Intervention group N = 130 patients	*p* Value
*Communication re patients*			
Patients with any type of medication communication *n* (%)	126 (96.9%)	124 (95.4%)	0.52^*^
Number of patients with allergy/ADR history discussed *n* (%)	48 (36.9%)	43 (33%)	0.54^*^
Number of patients whose adherence was discussed *n* (%)	19 (14.6%)	14 (10.8%)	0.36^*^
*Medication communication: in depth discussions*			
Number of patients who had an in-depth medication discussion for *n* (%)	100 (76.9%)	122 (93.8%)	< 0.001^*^
Number of in-depth medication discussions *n* (mean, SD)	249 (1.9 ± 1.7/patient)	352 (2.7 ± 1.7/patient)	< 0.001^†^
Number of in-depth medication discussions relating to high risk (APINCH) medications *n* (mean, SD)	92 (0.71 ± 1.1/patient)	156 (1.2 ± 1.2/patient)	< 0.001^†^
Proportion of in-depth discussions (actionable) which were actioned during PTWR *n* (%)	154/194 (79.4%)	236/284 (83.1%)	0.30^*^
Proportion of in-depth discussions (actionable) which were actioned on PTWR or inpatient stay *n* (%)	178/194 (91.7%)	264/284 (92.9%)	0.62^*^

Statistical tests: * Chi squared, † student’s *T*-test

When analysing the difference in APINCH classifications for the in-depth medication related discussions relating to high-risk medications, the proportional spread across APINCH categories showed no significant variation between the comparator and intervention group.

In line with the increased number of in-depth discussions, there was a statistically significantly higher number of in depth discussions actioned in the intervention group, compared with comparator, both at the end of the PTWR, and at discharge. However when expressed as a proportion of those able to be actioned, there was no significant difference in the proportion of in-depth discussions actioned during the PTWR in comparator and intervention group and no difference in the proportion actioned during the inpatient stay (see Table 2).

### Medication appropriateness (START/STOPP tool)

The overall proportion of patients who had an improvement in their START/STOPP scores was significantly higher for the intervention group with 48 patients (37%) compared to 33 patients (25%) in the comparator group (*p* = 0.004). There was a greater difference in the proportion of patients who had a START/STOPP score improvement for the sub-group of patients 65 years or older (intervention 42%, comparator 23%, *p* = 0.014).

**Fig. 2:**
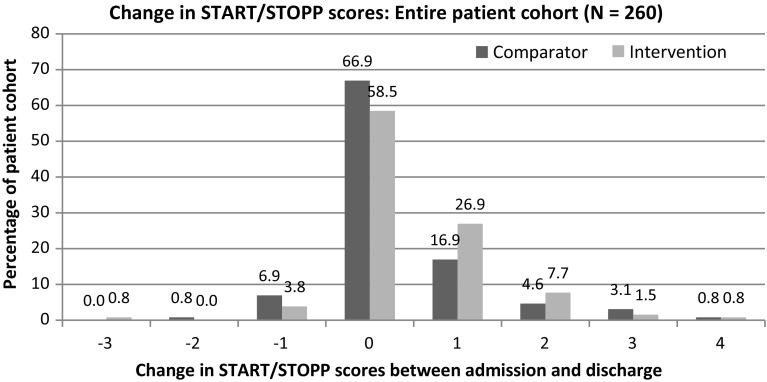
Change in START/STOPP scores: entire cohort

**Fig. 3:**
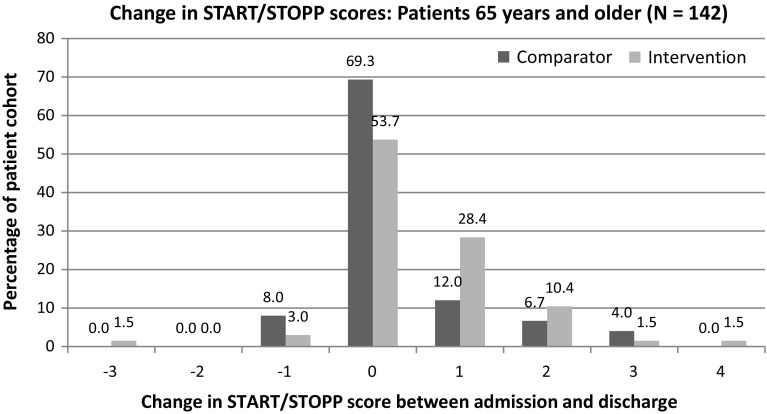
Change in START/STOPP scores: patients 65 years and older

Figure 2 illustrates the change in START/STOPP scores between admission and discharge across the entire patient cohort with changes ranging from a decrease in 3 START/STOPP criteria to an increase in 4 criteria. Figure 3 illustrates the frequency of START/STOPP changes for the patients over the age of 65 (Table 3).

**Table 3:** Medication appropriateness

	Comparator group (N = 130)	Intervention group (N = 130)	*p* Value
Patients with improvement in overall medication appropriateness—n (%)	33 (25%)	48 (37%)	0.004*
Medication appropriateness—mean ± SD			
*Admission*			
START	0.94 ± 1.16	1.08 ± 1.23	
STOPP	0.95 ± 1.02	0.78 ± 0.90	
Overall START/STOPP	1.89 ± 1.58	1.85 ± 1.56	
*Discharge*			
START	0.85 ± 1.06	0.85 ± 1.12	
STOPP	0.75 ± 0.83	0.56 ± 0.81	
Overall START/STOPP	1.59 ± 1.39	1.42 ± 1.43	
Difference overall START/STOPP admission to discharge	0.30 ± 0.87	0.43 ± 0.88	0.049

Statistical tests: * Chi squared, † student’s *T*-test

The mean change in START/STOPP scores between admission and discharge was significantly higher for the intervention group (0.43 ± 0.88) than for the comparator group (0.3 ± 0.87; *p* = 0.049). In patients 65 years or older, there was a greater improvement in medication appropriateness in the intervention group (0.52 ± 0.99) compared with the comparator group (0.3 ± 0.87; *p* = 0.028), or an extra 22 appropriate medications per 100 patient admissions.

### Patient and service delivery related outcomes

The ward-based clinical pharmacy services including patients who had a ward pharmacist review, the number who had a Medication Action Plan documented and who had a DMR prepared on discharge, length of stay and readmission rates were similar in the comparator and intervention periods (see Table 4).

**Table 4:** Patient and service delivery related outcomes

	Comparator group (N = 130)	Intervention group (N = 130)	*p* Value
Duration of the PTWR (e.g., time spent by PTWR team by with each patient) (mean, SD) *minutes*	23.8 ± 8.9	20.7 ± 9.9	< 0.008^†^
Ward pharmacist review (minimum of one pharmacy review by ward pharmacist during stay)	116 (89.2%)	108 (83%)	0.26^*^
Medication action plan (MAP) completed by pharmacist during admission	112 (86.2%)	104 (80%)	0.29^*^
Length of stay (days) *Median (IQR)*	4 (2–7)	4 (2–7)	0.34^†^
Discharge medication record (DMR) prepared for patient and patient counselled by clinical pharmacist	80 (62%)	73 (56.2%)	0.34^*^
Unplanned readmission < 30 days	15 (11.5%)	11 (8.5%)	0.43^*^

Statistical tests: * Chi squared, † student’s *T*-test

## Discussion

When a clinical pharmacist participated in the PTWR there was greater medication communication and an increase in the level of medication appropriateness at discharge compared to usual care. The mean change in medication appropriateness was small (improvement of 0.13 START/STOPP indicators per patient) however this is equivalent to 13 extra appropriate medications or the de-prescribing of 13 extra potentially inappropriate medications per 100 patients. There was an extra 12% of patients had an improvement in medication appropriateness, which equates to a number needed-to-treat of just over eight. Significant differences were seen in all patients and in the subgroup of patients aged 65 years or older, where the START/STOPP tool has been validated (number needed-to-treat for an improvement of medication inappropriateness of just over five) [18].

The model of care enacted in the intervention group allowed direct pharmacist input to prescribing discussion and decisions with senior members of the medical team during the PTWR, compared with usual care when ward pharmacists liaise with more junior medical staff after the senior medical team have made their input. We suggest that this high-level interaction, which led to an increase in both superficial and in-depth discussions related to medications, both generally and to high-risk medications resulted in improvements in medication appropriateness. These findings support the proposition by Fertleman et al. that clinical pharmacists influence decisions at the point of prescribing [2] and the evidence from Leape and Scarsi that pharmacist interventions can lead to reduced medication related errors ADEs [2].

Reassuringly, the presence of the pharmacist did not prolong the time spent with each patient. The time spent by the PTWR team with each patient across both study cohorts was longer than the average found in a study by Herring et al. [19] which found an average time per patient of 12 min (10 min on routine rounds and 14 min on post-take rounds).

There were a significantly higher absolute number of in-depth discussions with agreed medication actions to be enacted in the intervention group. The proportion of those not actioned, however, did not significantly decrease with the presence of a PTWR pharmacist in the intervention group. This highlights further opportunity to optimise medications though appropriate documentation of the medication plan during the PTWR, improved handover from the PTWR pharmacist and follow up by, the ward based pharmacist to ensure agreed decisions are actioned.

In comparison to the previous work in this area [2], there was no significant difference found in the change in number of medications in the two groups from admission to discharge. However, we demonstrated an overall improvement in medication appropriateness which may indicate that any reduction in inappropriately prescribed medication was offset by an increase in medication that was not previously prescribed, but should have been.

We believe that our study results are generalizable to similar general medical units for various reasons. Firstly, the clinical pharmacists involved in the PTWRs required well-developed clinical knowledge, communication and interpersonal skills in order to actively engage in the ward round [6] and hence a minimum level of experience and competency was defined for the pharmacists attending PTWRs. It was felt that this level of experience would allow individuals to think proactively in the fast paced environment, hold a high level of clinical reasoning and develop good working relationships with medical staff. We believe that using four different pharmacists, with this minimum skill set, improves the generalisability of our results which has not previously been achieved in studies of this type [2]. According to coding data, it was felt that the study cohort was representative of a normal general medicine cohort. Lastly, patients under the care of all eight of the hospitals internal medical teams were included in both phases of this study and observations of all levels of medical staff as part of those teams was undertaken.

### Limitations

Evidence from controlled studies demonstrates enhanced medication liaison reduces unplanned admissions to hospital [10]. In our study, a significant limitation was that close to 40% (153/260) of all patients across the comparator and intervention groups did not receive a pharmacist generated DMR when they left the hospital to guide them and their primary care team in the continuation of what was, for the intervention group, a significantly more appropriate medication regime at the time of discharge. This highlights a significant gap in medication handover between the hospital and primary care team with a high risk that some improvements in medication appropriateness made were not handed over to the patient or their general practitioner on discharge.

A further limitation of this study is that the comparator and intervention observations were not carried out at the same time of the year and therefore other changes within the hospital, such as staff changes due to junior doctor rotations, may have impacted on outcomes. However, the observation periods were chosen to minimize this limitation with a six-week gap in between to reduce compounding factors.

In addition, the lead researcher was not completely independent. Observation bias was minimised by having the same observer recording data for both the comparator and intervention groups using a standardized data collection method and tool.

Our study did not demonstrate any statistically significant changes in patient and Service Delivery Related Outcomes (e.g., length of stay, unplanned readmissions), we did not collect data related to mortality and follow up was short (30 days). However, our study was not powered to detect differences in these outcomes. The results of this study could be used to estimate a sample size for a larger, more definitive trial.

At the time of study observation, the current START/STOPP tool Version 1 was applied. Since observation completion, an updated version 2 of the START/STOPP criteria has been published. Of note, version 2 of START/STOPP, with 114 criteria, represents a 31% increase in the total number of criteria included in version 1. The improvements found in medication appropriateness in our study as measured by the START/STOPP criteria version 1 may therefore be an underestimation of those found if the updated criteria were applied.

### Implication on practice and future research

This paper focuses on medication communication and appropriateness outcomes for patients and its findings have potential impacts on patients, professionals and the health care system. The findings demonstrate that interdisciplinary input to decision making can improve the appropriateness of medications for patients and outline the scope for clinical pharmacist input to improve collaborative optimisation of medication use and ensure that decisions made by medical staff around medication prescribing are handed over, followed through and enacted. This model of care ensures the appropriate use of health professional skill mix by ensuring Pharmacists are used at the optimal time for patient care input.

The cost of medication errors and inappropriate medications is high and the findings of improvement in medication appropriateness and communication in this study warrant further economic evaluation to assess whether a PTWR pharmacist service is cost-effective and possibly cost saving to the health system.

## Conclusion

Clinical pharmacist participation on the PTWR leads to an increased level of medication-related communication and discussions targeted at high-risk medications, increased opportunity for collaborative decision making and improved medication appropriateness for patients and is therefore is a worthwhile endeavour. This provides further evidence on this alternative model of service delivery that is consistent with benefits seen in other studies in this area.
